# Supplementary material for: HIVprotI: an integrated web based platform for prediction and design of HIV proteins inhibitors
Source: J Cheminform. 2018 Mar 9;10:12. doi: 10.1186/s13321-018-0266-y (PMC5845081; doi:10.1186/s13321-018-0266-y)
Supplement: Supplementary file 2 — Additional file 2. Source code of HIVProtI web server. [file 13321_2018_266_MOESM2_ESM.zip › HIVprotI_Source-code/hivprotI/weka-3-6-6/doc/weka/core/neighboursearch/kdtrees/KDTreeNode.html]

KDTreeNode


---


|  |  |  |
| --- | --- | --- |
| **Overview** \| **Package** \| **Class** \| **Tree** \| **Deprecated** \| **Index** \| **Help** \| **Weka's home** | |  |
| PREV CLASS   **NEXT CLASS** | **FRAMES**    **NO FRAMES**     **All Classes** |  |
| SUMMARY: NESTED \| FIELD \| CONSTR \| METHOD | DETAIL: FIELD \| CONSTR \| METHOD |  |


---


## weka.core.neighboursearch.kdtrees Class KDTreeNode

```
java.lang.Object
  weka.core.neighboursearch.kdtrees.KDTreeNode
```

**All Implemented Interfaces:**: java.io.Serializable, RevisionHandler

---

``` public class KDTreeNode extends java.lang.Object implements java.io.Serializable, RevisionHandler ```

A class representing a KDTree node. A node does not explicitly
store the instances that it contains. Instead, it only stores
the start and end index of a portion in a master index array. Each
node is assigned a portion in the master index array that stores
the indices of the instances that the node contains. Every time a
node is split by the KDTree's contruction method, the instances of
its left child are moved to the left and the instances of its
right child are moved to the right, in the portion of the master
index array belonging to the node. The start and end index in each
of its children are then set accordingly within that portion so
that each have their own portion which contains their instances.
P.S.: The master index array is only stored in KDTree class.

**Version:**
:   $Revision: 1.2 $

**Author:**
:   Ashraf M. Kibriya (amk14[at-the-rate]cs[dot]waikato[dot]ac[dot]nz)

**See Also:**: Serialized Form

---

| **Field Summary** | |
| --- | --- |
| `int` | `m_End`             The end index of the portion of the master index array, which stores indices of the instances/points the node contains. |
| `KDTreeNode` | `m_Left`             left subtree; contains instances with smaller or equal to split value. |
| `int` | `m_NodeNumber`             node number (only for debug). |
| `double[][]` | `m_NodeRanges`             lowest and highest value and width (= high - low) for each dimension. |
| `double[][]` | `m_NodesRectBounds`             The lo and high bounds of the hyper rectangle described by the node. |
| `KDTreeNode` | `m_Right`             right subtree; contains instances with larger than split value. |
| `int` | `m_SplitDim`             attribute to split on. |
| `double` | `m_SplitValue`             value to split on. |
| `int` | `m_Start`             The start index of the portion of the master index array, which stores the indices of the instances/points the node contains. |


**Constructor Summary**

`KDTreeNode()`
  
          Constructor.

`KDTreeNode(int nodeNum,
int startidx,
int endidx,
double[][] nodeRanges)`
  
          Constructor.

`KDTreeNode(int nodeNum,
int startidx,
int endidx,
double[][] nodeRanges,
double[][] rectBounds)`


| **Method Summary** | |
| --- | --- |
| `java.lang.String` | `getRevision()`             Returns the revision string. |
| `int` | `getSplitDim()`             Gets the splitting dimension. |
| `double` | `getSplitValue()`             Gets the splitting value. |
| `boolean` | `isALeaf()`             Checks if node is a leaf. |
| `int` | `numInstances()`             Returns the number of Instances in the rectangular region defined by this node. |

**Methods inherited from class java.lang.Object**

`equals, getClass, hashCode, notify, notifyAll, toString, wait, wait, wait`

**Field Detail**

### m\_NodeNumber

```
public int m_NodeNumber
```

:   node number (only for debug).

---


### m\_Left

```
public KDTreeNode m_Left
```

:   left subtree; contains instances with smaller or equal to split value.

---


### m\_Right

```
public KDTreeNode m_Right
```

:   right subtree; contains instances with larger than split value.

---


### m\_SplitValue

```
public double m_SplitValue
```

:   value to split on.

---


### m\_SplitDim

```
public int m_SplitDim
```

:   attribute to split on.

---


### m\_NodeRanges

```
public double[][] m_NodeRanges
```

:   lowest and highest value and width (= high - low) for each
    dimension.

---


### m\_NodesRectBounds

```
public double[][] m_NodesRectBounds
```

:   The lo and high bounds of the hyper rectangle described by the
    node.

---


### m\_Start

```
public int m_Start
```

:   The start index of the portion of the master index array,
    which stores the indices of the instances/points the node
    contains.

---


### m\_End

```
public int m_End
```

:   The end index of the portion of the master index array,
    which stores indices of the instances/points the node
    contains.


**Constructor Detail**

### KDTreeNode

```
public KDTreeNode()
```

:   Constructor.

---


### KDTreeNode

```
public KDTreeNode(int nodeNum,
                  int startidx,
                  int endidx,
                  double[][] nodeRanges)
```

:   Constructor.

    **Parameters:**: `nodeNum` - The node number/id.: `startidx` - The start index of node's portion in master index array.: `endidx` - The start index of node's portion in master index array.: `nodeRanges` - The attribute ranges of the Instances/points contained in this node.

---


### KDTreeNode

```
public KDTreeNode(int nodeNum,
                  int startidx,
                  int endidx,
                  double[][] nodeRanges,
                  double[][] rectBounds)
```

**Parameters:**: `nodeNum` - The node number/id.: `startidx` - The start index of node's portion in master index array.: `endidx` - The start index of node's portion in master index array.: `nodeRanges` - The attribute ranges of the Instances/points contained in this node.: `rectBounds` - The range of the rectangular region in the point space that this node represents (points inside this rectangular region can have different range).


**Method Detail**

### getSplitDim

```
public int getSplitDim()
```

:   Gets the splitting dimension.

    :   **Returns:**: splitting dimension

---


### getSplitValue

```
public double getSplitValue()
```

:   Gets the splitting value.

    :   **Returns:**: splitting value

---


### isALeaf

```
public boolean isALeaf()
```

:   Checks if node is a leaf.

    :   **Returns:**: true if it is a leaf

---


### numInstances

```
public int numInstances()
```

:   Returns the number of Instances
    in the rectangular region defined
    by this node.

    :   **Returns:**: The number of instances in this KDTreeNode.

---


### getRevision

```
public java.lang.String getRevision()
```

:   Returns the revision string.

    :   **Specified by:**: `getRevision` in interface `RevisionHandler`
    :   **Returns:**: the revision


---


|  |  |  |
| --- | --- | --- |
| **Overview** \| **Package** \| **Class** \| **Tree** \| **Deprecated** \| **Index** \| **Help** \| **Weka's home** | |  |
| PREV CLASS   **NEXT CLASS** | **FRAMES**    **NO FRAMES**     **All Classes** |  |
| SUMMARY: NESTED \| FIELD \| CONSTR \| METHOD | DETAIL: FIELD \| CONSTR \| METHOD |  |


---
